# Pituitary adenylate cyclase-activating polypeptide mediates bacterial endotoxin-induced fever via an effect on cyclooxygenase-2 and inflammatory cytokines

**DOI:** 10.1038/s41598-025-08352-0

**Published:** 2025-07-03

**Authors:** Jason Sparks, Nora Furedi, Kata Fekete, Margit Solymar, Krisztina Pohoczky, Agnes Kemeny, Dora Reglodi, Andras Garami, Eszter Pakai

**Affiliations:** 1 Hungarian Research Network (HUN-REN-PTE), PACAP Research Group, Department of Anatomy, Medical School, University of Pecs, Pecs, Hungary; 2 Department of Anatomy, Medical School, University of Pecs, Pecs, Hungary; 3 Department of Thermophysiology, Institute for Translational Medicine, Medical School, University of Pecs, Pecs, Hungary; 4 Department of Pharmacology, Faculty of Pharmacy, University of Pecs, Pecs, Hungary; 5 Department of Pharmacology and Pharmacotherapy, Medical School, University of Pecs, Pecs, Hungary; 6 Hungarian Research Network (HUN-REN-PTE), Chronic Pain Research Group, University of Pecs, Pecs, Hungary; 7 Department of Physiology and Biochemistry, University of Veterinary Medicine, Budapest, Hungary

**Keywords:** PACAP, Thermoregulation, Fever, LPS, COX-2, Systemic inflammation, Endocrine system and metabolic diseases, Inflammation, Fever, Interleukins

## Abstract

A role for pituitary adenylate cyclase-activating polypeptide (PACAP) signaling was suggested in bacterial lipopolysaccharide (LPS)-induced fever, but the underlying mechanisms of how PACAP contributes to the febrile response have remained unclarified. We administered LPS (120 µg/kg, intraperitoneally) to mice with the *Pacap* gene either present (*Pacap*^+*/*+^) or absent (*Pacap*^*−/−*^) and measured their thermoregulatory responses, serum cytokine levels, and tissue cyclooxygenase-2 (COX-2) expression. LPS-induced fever was attenuated in *Pacap*^*−/−*^ mice compared to their *Pacap*^+*/*+^ littermates from ~ 120 min postinfusion. LPS increased COX-2 mRNA expression in the lungs, liver, and brain in *Pacap*^+*/*+^ mice at 210 min postinfusion. In the LPS-treated groups, COX-2 mRNA upregulation in *Pacap*^*−/−*^ mice was attenuated in the liver, but augmented in the lungs and brain compared to *Pacap*^+*/*+^ mice. In response to LPS, serum concentrations of interleukin (IL)-1α and β were markedly increased in *Pacap*^+*/*+^ mice, but not in *Pacap*^*−/−*^ mice, with a significant intergenotype difference between the groups. Serum concentrations of IL-6, IL-10, and TNF-α were higher after LPS treatment compared to saline in both genotypes, however, the rise in IL-10 was significantly attenuted in *Pacap*^*−/−*^ mice compared to *Pacap*^+*/*+^ mice. We showed that PACAP contributes to the later phases of LPS-induced fever by modulation of COX-2 expression in the periphery and the brain, as well as by augmentation of circulatory pyrogenic cytokine levels. These findings advance the understanding of the crosstalk between PACAP signaling and the “cytokine-COX-2” axis in systemic inflammation.

## Introduction

Pituitary adenylate cyclase-activating polypeptide (PACAP) is a multifaceted neuroendocrinological mediator, which was originally isolated from the hypothalamus^1,2^, but it turned out that it is produced by a variety of tissues and cell types, including neurons and immune cells^3^. The role of PACAP is established in different homeostasis processes such as nociception, inflammation, energy balance, and thermoregulation^4–7^.

Systemic inflammation is often induced in experimental models by the administration of bacterial lipopolysaccharide (LPS). High doses of LPS (several mg/kg) cause serious, often lethal systemic inflammation, which is typically accompanied by a decrease in deep body temperature (T_b_)^8^. In such severe forms of systemic inflammation, PACAP exerted a protective effect in mice and dogs^9,10^. In addition, the protective role of PACAP signaling was also demonstrated in LPS-induced shock with mice genetically lacking the specific PACAP receptor PAC1^11^. An interaction between PACAP signaling and pro-inflammatory cytokines, such as interleukin (IL)-6 and tumor necrosis factor (TNF)-α, was also shown in such experimental models^9,11,12^.

In contrast to severe systemic inflammation, mild-to-moderate systemic inflammatory response can be triggered by lower doses of LPS (often in the µg/kg range), which is characterized by fever. The febrile response is mediated by pyrogenic cytokines (IL-1, -6, and TNF-α) and by the activation of the arachidonic acid (AA) pathway, wherein cyclooxygenase (COX) enzyme activity leads to production of prostaglandin E_2_ (PGE_2_)^13,14^. In the median preoptic nucleus (MnPO), PGE_2_ activates thermoregulatory neurons, which leads to the rise of deep T_b_ via increased thermogenesis and skin vasoconstriction^8^. In addition to this well-established molecular pathway, neuroendocrinological mediators, for example, substance P and cholecystokinin signaling, have also been identified as contributors to the fever response^15,16^. Interestingly, the intracerebral administration of PACAP to rats resulted in increased oxygen consumption (thermogenesis) and skin vasoconstriction in rats^17^, which is similar to the thermoeffector pattern of febrigenesis. Moreover, the PACAP-induced increase in T_b_ could be attenuated by COX enzyme inhibition^18^, indicating an interaction between PACAP signaling and the COX pathway. However, the potential role of PACAP in the development of the LPS-induced fever response has not been investigated despite its complex function in homeostasis, which entails both pro- and anti-inflammatory roles^11,19–21^.

In the present work, we studied how the genetic ablation of PACAP influences the LPS-induced early and later phases of the fever response by comparing mice, which had the *Pacap* gene homozygously either present (*Pacap*^+*/*+^) or absent (*Pacap*^*-/-*^) due to a targeted disruption^22^. In thermophysiological experiments, we recorded changes in deep T_b_ in response to LPS. To identify the involved molecular mechanisms, we measured serum cytokine levels, as well as tissue COX-2 mRNA expression in this animal model.

## Results

### LPS-induced changes in the thermoregulatory response of ***Pacap***^+***/***+^ and ***Pacap***^***-/-***^ mice

**Fig. 1:**
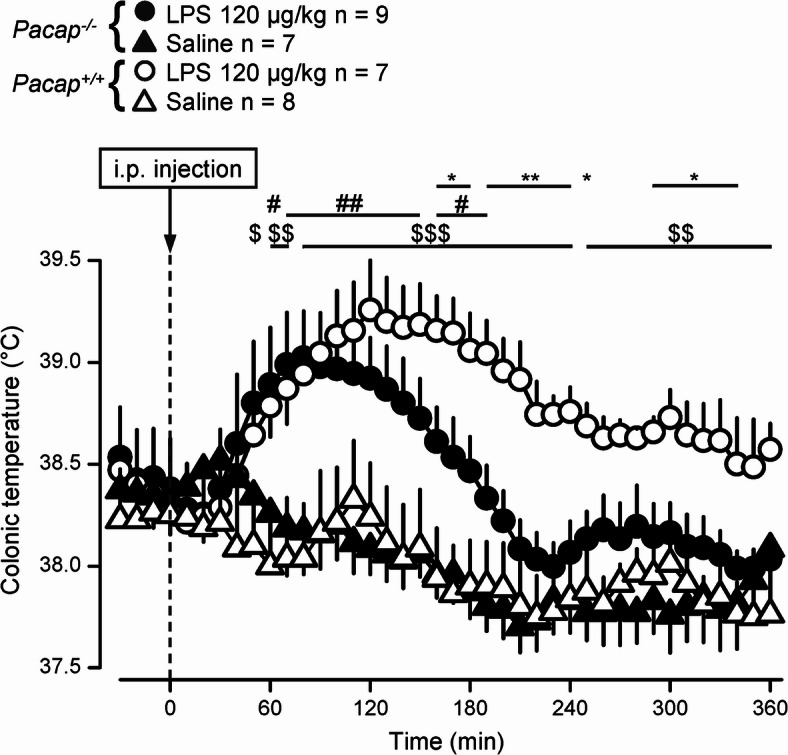
Changes in deep (colonic) T_b_ in response to i.p. administration of LPS (120 µg/kg) or saline in *Pacap*^+*/*+^ and *Pacap*^*-/-*^ mice. *p < 0.05, **p < 0.01 intergenotype difference in LPS-treated mice; ^#^*p* < 0.05, ^##^*p* < 0.01 difference between treatments in *Pacap*^*-/-*^ mice; ^$^*p* < 0.05, ^$$^*p* < 0.01, ^$$$^*p* < 0.001 difference between treatments in *Pacap*^+*/*+^ mice.

To compare the fever response between *Pacap*^+*/*+^ and *Pacap*^*-/-*^ mice, we administered LPS [120 µg/kg, intraperitoneally (i.p.)] or saline to the mice of both genotypes. The infusion of saline did not cause any effect on deep T_b_ in either genotype, as expected based on our previous study^16^. On the contrary, LPS-treated mice developed fever as compared to their saline-treated counterparts (Fig. 1). In *Pacap*^+*/*+^ mice LPS caused a characteristic fever response: their deep T_b_ started to increase at 40 min, plateaued (~ 39.1 °C) between 100 and 170 min postinfusion, then it gradually decreased, but remained elevated compared to saline treatment throughout the experiment (*p* < 0.05 at 50–360 min). These findings are in agreement with those reported in genetically unmodified mice in previous studies^16,23^. However, in *Pacap*^*-/-*^ mice, the LPS-induced fever response was less pronounced than in their *Pacap*^+*/*+^ littermates: their T_b_ reached plateau at 60 min (~ 38.9 °C), but started to decrease earlier (at 140 min) and returned to the level of saline-treated mice already at 200 min post-LPS administration (Fig. 1). Statistically, the intergenotype difference between the LPS-treated groups was significant at 160–250 min and 290–340 min postinfusion (*p* < 0.05). Importantly, the T_b_ of the LPS-treated *Pacap*^*-/-*^ mice was markedly (0.5–0.8 °C) lower than that of *Pacap*^+*/*+^ mice starting from 160 min post-LPS infusion until the end of the experiment (*p* < 0.05).

### LPS-induced changes in COX-2 mRNA expression in the lungs, liver, and brain of ***Pacap***^+***/***+^ and ***Pacap***^***-/-***^ mice

**Fig. 2:**
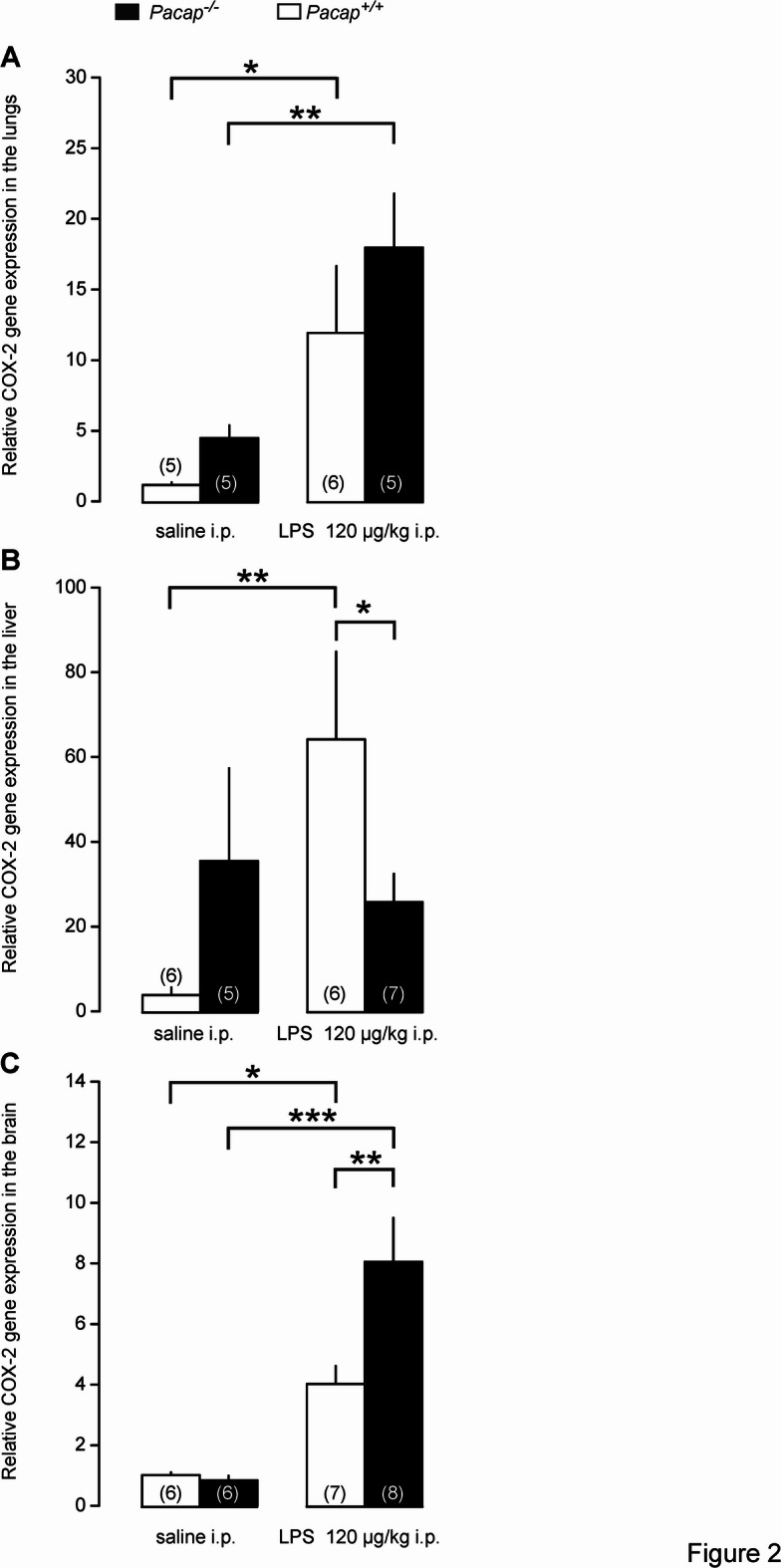
Relative COX-2 gene expression in the lungs (**A**), liver (**B**), and brain (**C**) of *Pacap*^+*/*+^ and *Pacap*^*-/-*^ mice after infusion of LPS (120 µg/kg) or saline. Tissue samples were collected at 210 min postinfusion. Number of animals in the corresponding groups are indicated in the figure. Significant differences are marked as *p < 0.05, **p < 0.01, ***p < 0.001.

Next, we compared the LPS-induced COX-2 expression in peripheral LPS-processing organs (i.e., lungs and liver) and in the brain between *Pacap*^+*/*+^ and *Pacap*^*-/-*^ mice. For that, lung, liver, and brain samples were collected at 210 min post-LPS infusion, i.e., at the time point at which the biggest difference in T_b_ was found between the genotypes (Fig. 1). In *Pacap*^+*/*+^ mice, LPS induced a significant increase in COX-2 expression in the lungs (Fig. 2A), liver (Fig. 2B), and brain (Fig. 2C), consistent with findings from previous studies in genetically unmodified rodents^16,24,25^. In *Pacap*^*-/-*^ mice, the LPS-induced increase in COX-2 expression was also significant compared to saline treatment in the lungs and the brain, but not in the liver (Fig. 2). Importantly, we found a significant difference in COX-2 mRNA expression between LPS-treated *Pacap*^+*/*+^ and *Pacap*^*-/-*^ mice: in *Pacap*^*-/-*^ mice, the LPS-induced COX-2 mRNA expression was significantly reduced in the liver (Fig. 2B), whereas it was more pronouncedly elevated in the brain compared to *Pacap*^+*/*+^ mice (Fig. 2C).

### LPS-induced changes in serum cytokine levels of ***Pacap***^+***/***+^ and ***Pacap***^***-/-***^ mice

**Fig. 3:**
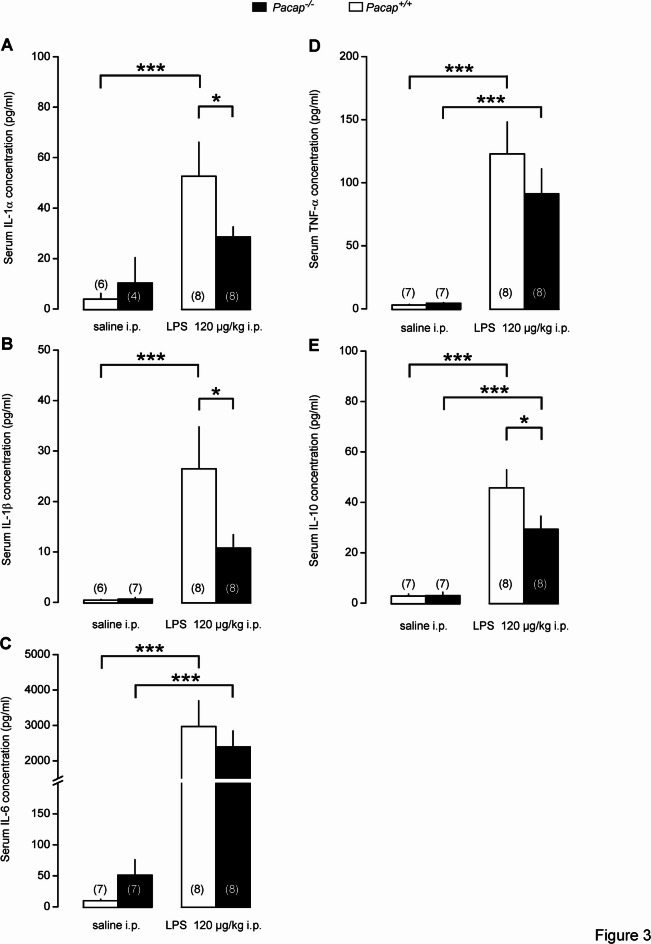
Serum cytokine concentrations in *Pacap*^+*/*+^ and *Pacap*^*-/-*^ mice. Serum IL-1α (**A**), IL-1β (**B**), TNF-α (**C**), IL-6 (**D**), and IL-10 (**E**) concentrations in *Pacap*^+*/*+^ and *Pacap*^*-/-*^ mice in response to LPS (120 µg/kg) or saline. Blood samples were collected at 210 min postinfusion. Number of animals in the corresponding groups are indicated in the figure. Significant differences are marked as *p < 0.05, ***p < 0.001.

The serum concentrations of the pyrogenic cytokines IL-1α and -β were significantly increased in *Pacap*^+*/*+^ mice in response to LPS compared to their saline-treated counterparts (*p* < 0.001 for both cytokines), whereas in *Pacap*^*-/-*^ mice LPS did not cause a significant change in their levels compared to saline treatment (Fig. 3A and B). The intergenotype difference between the LPS-treated groups was also significant for both IL-1α and -β (*p* < 0.05 for both cytokines). In cases of IL-6 and TNF-α, the administration of LPS resulted in a marked (*p* < 0.001) rise in both genotypes, while saline had no meaningful effect (Fig. 3C and D). The levels of the anti-inflammatory IL-10 were higher in LPS-treated than in saline-treated mice in both genotypes (*p* < 0.001), however the rise was significantly attenuated in *Pacap*^*-/-*^ mice compared to their *Pacap*^+*/*+^ littermates (*p* < 0.05) (Fig. 3E).

## Discussion

The present study provides novel evidence demonstrating that the fever response to bacterial endotoxin is significantly attenuated in *Pacap*^*-/-*^ mice. We also found that the LPS-induced COX-2 mRNA upregulation was reduced in the liver, whereas increased in the brain of *Pacap*^*-/-*^ mice compared to their *Pacap*^+*/*+^ littermates. Furthermore, the blood levels of the pyrogenic cytokines IL-1α and -β, as well as the level of the anti-inflammatory cytokine IL-10 were decreased, while levels of the pro-inflammatory IL-6 and TNF-α tended to be lower in the absence of PACAP in response to LPS.

Systemic inflammation involves a balance between pro- and anti-inflammatory processes^26^: the systemic inflammatory response (SIR) is counteracted by a compensatory anti-inflammatory (CAR) response^27^. Systemic inflammation is often associated with changes in deep T_b_, most commonly with fever that is a thermoregulatory response of the host organism to facilitate the elimination of the pathogens^8^. In experimental models, systemic inflammation-associated fever is often induced with the administration of the bacterial endotoxin LPS.

**Fig. 4:**
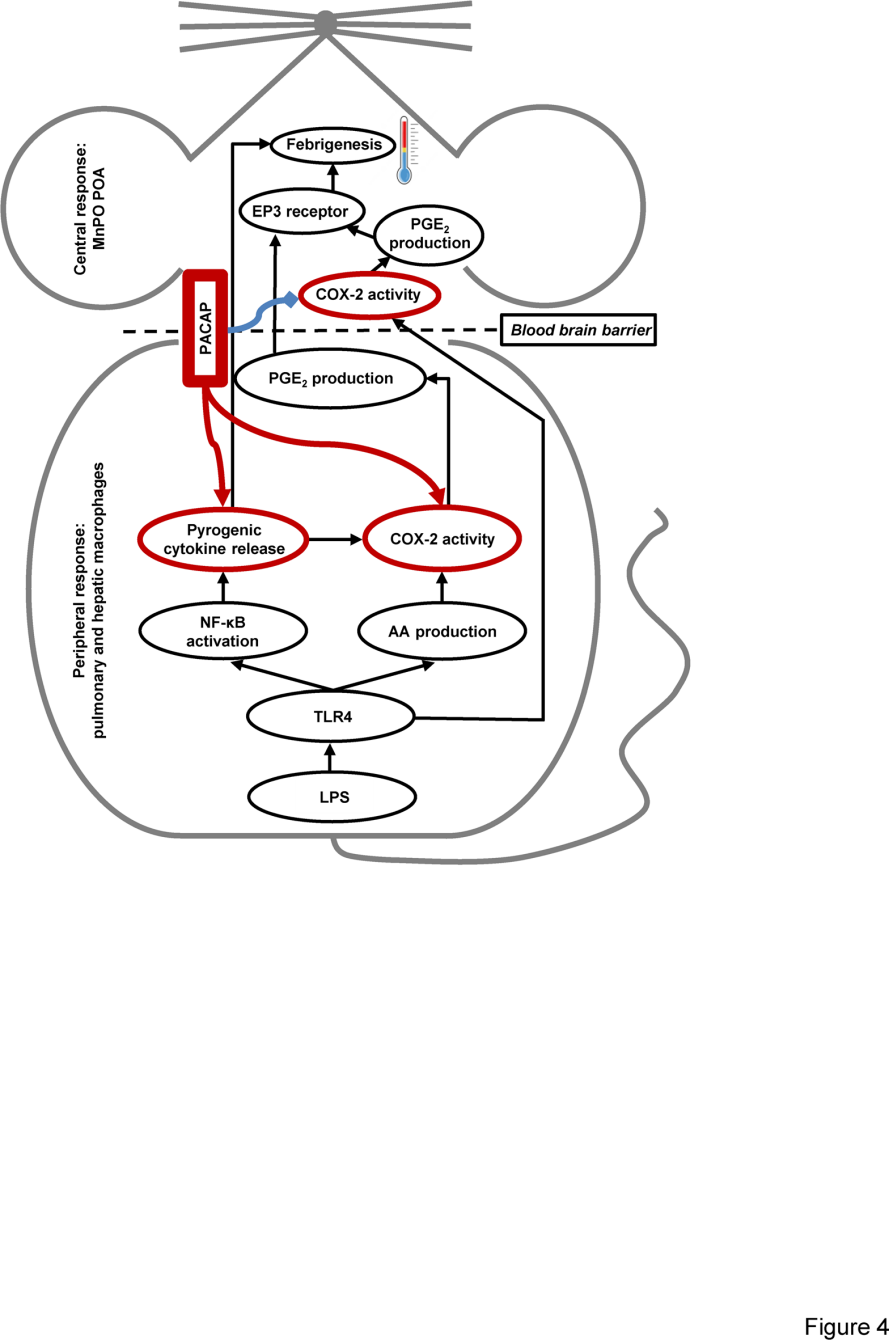
Schematic representation of febrigenesis and the potential influence of PACAP. For explanation, see text. POA, preoptic area of the hypothalamus.

PGE_2_ production in peripheral organs such as the lungs and liver contribute to the initial phases of LPS-induced fever, while later phases are maintained by production of PGE_2_ within the brain^8,28,29^. It should be also mentioned that by using genetically modified mice, Shionoya et al.^30^ showed that PGE_2_ synthesis in brain endothelial cells is responsible for all phases of fever, which challenges the importance of peripheral organs in febrigenesis. In the early phase, LPS via binding to toll-like receptor 4 (TLR4), activates the AA pathway involving the upregulation of COX-2, as well as the increased production of pro-inflammatory (pyrogenic) cytokines such as IL-1, -6, and TNF-α via nuclear translocation of nuclear factor kappa B (NF-κB). Within the brain, PGE_2_ binds to prostaglandin E type 3 (EP3) receptor expressed on thermoregulatory neurons in the MnPO of the hypothalamus, which in turn leads to the elevation of T_b_^8,29,31^. While the aforementioned canonical mechanisms of fever are well established (for reviews, see^8,29,32^), novel mediators, which can influence the activities of the classical pathways are often discovered^15,16,33,34^. A schematic of the described mechanisms of febrigenesis, also including how they can be influenced by PACAP based on the findings of the current study, are presented in Fig. 4.

It has been demonstrated by several research groups that PACAP plays an important role in thermoregulation and contributes to the defense of energy homeostasis (for reviews, see^4,35^). It was also suggested that PACAP contributes to the LPS-induced systemic inflammation in dogs^10^, mice^9,11,21^, and rats^12^. However, it has remained unclear until now, whether and how PACAP may play a role in the development of the LPS-induced elevation of deep T_b_. Here, we showed that LPS-induced fever was attenuated in the absence of PACAP starting from ~ 120 min post-LPS infusion, which clearly indicates that PACAP is required for the febrile response. This finding is in harmony with our previous results about the fever-like thermoregulatory response to PACAP administration in rats and the decreased basal T_b_ and metabolic rate in *Pacap*^*-/-*^ mice presumably caused by enhanced suppression of thermogenesis by neurons in the medial preoptic area^17^.

To explore the molecular mechanism in the background of the attenuated fever response in the absence of PACAP, we studied the expression of COX-2 in peripheral LPS-processing organs (i.e., lungs and liver) and in the brain. We showed that in the absence of PACAP the LPS-induced upregulation of COX-2 was attenuated in the liver, whereas it was augmented in the brain. The reduced hepatic COX-2 upregulation can well explain the attenuated fever response to LPS in the absence of PACAP (see Fig. 4). In our previous study, we found a similarly reduced fever and attenuated COX-2 upregulation in peripheral organs in response to LPS in the genetic absence of the neurokinin-1 receptor^16^, which is a key receptor in substance P signaling^36^. Interestingly, a close interaction between PACAP and substance P pathways in inflammatory processes has been demonstrated in multiple studies^37–39^. Hence, it is plausible to assume that PACAP and substance P interact within the pyrogenic pathways of febrigenesis. It should be also mentioned that PACAP was recently shown to stimulate neuroendocrine and behavioral stress responses through corticotropin-releasing factor-containing neurons in the hypothalamus^40^. From human studies and animal experiments it is also known that LPS stimulates the hypothalamic–pituitary–adrenal axis, which is thought to be an important adaptive immunoregulatory response^41^. Since corticotropin-releasing factor plays an important role in the regulation of COX-2 under inflammatory conditions^42^, it may be assumed that an interaction between PACAP and the hypothalamic–pituitary–adrenal axis could have also contributed to our findings.

The augmented COX-2 upregulation induced by LPS in the brain of the *Pacap*^*-/-*^ mice was an unexpected finding. Different studies indicated that PACAP receptors are widely expressed throughout the cranial vasculature (for review, see^43^). In the central nervous system, the major source of PGE_2_ is the brain endothelium^8,29,32^. It was shown that PACAP inhibits the COX pathway of rat cerebral microvessels^44^, which may present a direct interaction between PACAP and COX-2 in brain endothelial cells to explain the higher LPS-induced COX-2 expression in the *Pacap*^*-/-*^ mice observed in our study. As an alternative explanation, microglial cells are among the main source of inflammatory mediators in the brain. It has been suggested that PACAP may exert its effects in part by acting directly on microglial cells as a microglia-deactivating factor^45,46^, which may lead to enhanced production of pro-inflammatory mediators, including PGE_2_, in the absence of PACAP. Since increased COX-2 expression is typically associated with higher PGE_2_ levels, a more pronounced febrile response could be expected. It is possible, however, that in the absence of PACAP downstream mediators in the AA cascade do not function properly, which could explain a compensatory, yet still ineffective, overexpression of COX-2 in the brain. In support of this scenario, it was shown that the majority of EP3 receptor-expressing neurons in the MnPO, which play a key role in the mediation of LPS-induced fever, express PACAP^47^. However, in that study it remained unanswered whether the release of PACAP is necessary to induce LPS fever. Based on our findings, it is plausible that the malfunction of these neurons in the absence of PACAP could contribute to the attenuated fever response even when COX-2 expression was higher. However, the discovery of the exact mechanism of the PACAP-COX-2 interaction in the brain was beyond the scope of the present study, thus remains subject of future research.

Pro-inflammatory cytokines, like IL-1α, -β, IL-6, and TNF-α, are crucial for triggering the immune response against pathogens. Nevertheless, anti-inflammatory cytokines, such as IL-10, also have an important role in limiting inflammation and in preventing tissue damage of the host^48,49^. Since pro- and anti-inflammatory cytokines play a pivotal role in the LPS-induced fever signaling pathway, we measured serum concentrations of these cytokines to assess whether the LPS-induced cytokine production is suppressed in the absence of PACAP. We found that in *Pacap*^*-/-*^ mice the LPS-induced elevation of IL-1α, -β, and IL-10 were significantly reduced, while that of IL-6 and TNF-α tended to be lower than in control mice. Since IL-1, -6, and TNF-α are well-documented pyrogenic cytokines (for review, see^8,32^), the smaller increase in their blood levels in response to LPS can explain the attenuation of the fever response in the absence of PACAP. It should be also noted that Mota and Madden^50^ showed that circulating IL-1β can elicit fever by activating brain circuits in the absence of PGE_2_ production, which raises the possibility for a role of PACAP in COX-independent febrigenesis.

The decreased levels of IL-10 in response to LPS in the absence of PACAP was an unexpected finding, since IL-10 has an antipyretic role in LPS fever^51,52^. However, taking into consideration the mitigated elevations of COX-2, pyrogenic cytokine levels, and deep T_b_, which are constituents of SIR, the smaller increase in anti-inflammatory mediators as part of the CAR can be expected. Thus, our findings suggest that PACAP is required for the production of the canonical pyrogenic cytokines in fever, but it also contributes to the regulation of the anti-inflammatory response, thereby maintaining the SIR-CAR balance in systemic inflammation. When PACAP is absent, the alteration in the pro- and anti-inflammatory cytokine levels as well as in the activity of AA cascade leads to the dysregulation of the SIR-CAR balance leading to a weakened fever response (Fig. 4).

Limitations of our study should be also mentioned. Fever signaling was examined only at a single time point in the current experiments due to the explorative nature of the study. It would be also interesting to see temporal changes of COX-2 expression and cytokine levels in the *Pacap*^*-/-*^ mice, since the kinetics of enzymes and cytokines incorporate time-dependent changes of their synthesis, metabolism, and elimination, which can show variations during systemic inflammation. Our results can serve as an encouraging basis for designing future studies, which aim to discover the kinetics of the inflammatory mediators in the absence of PACAP. Further, the use of constitutive PACAP knockout mice did not allow us to functionally analyze the site(s) of action of PACAP. An elegant method to discover the sites of action would be to use organ or tissue specific modulation of PACAP expression. For example, by using conditional PACAP deletion in their recent study, Bakalar et al.^53^ could distinguish between neurotransmitter and non-neurotransmitter functions of PACAP, including thermoregulatory. They showed that restriction of PACAP deletion to the hypothalamus prevents the thermoregulatory changes observed in constitutive *Pacap*^*-/-*^ mice, however hypothalamic PACAP-expressing neurons did not require PACAP for fasting-induced hypothermia. Similar animal models could also advance our knowledge regarding the specific site of action of PACAP in systemic inflammation-associated fever, but that was beyond the scope of the present study.

In conclusion, the modulation of COX-2 expression, as well as pro- and anti-inflammatory cytokine concentrations, by PACAP is required for the development of the fever response associated with systemic inflammation.

## Materials and methods

### Animals

Experiments were conducted in 42 *Pacap*^+*/*+^ and *Pacap*^*-/-*^ adult mice of both sexes. As in earlier studies^17,54,55^, the mice were obtained from the Animal Facility of Medical School, University of Pecs. Generation by a gene-targeting technique, maintenance, and backcrossing of PACAP deficient mice on a CD1 background has been described previously^22,56^. Animals were housed in temperature-controlled rooms on a 12 h light–dark cycle. Standard rodent chow and tap water were available *ad libitum*. At the time of the experiments, the mice weighed 37.2 ± 0.8 g.

For thermophysiological experiments mice were extensively handled (5 min per day for 8 days) and then habituated to the experimental setup as follows. Each mouse was adapted to staying in a wire-mesh confiner every day for gradually increasing time intervals (8 training sessions, 1–4 h each), as in previous studies^57,58^. The cylindrical confiner restricted the animal from turning around but permitted limited forward and backward movement. Small animals are readily adaptable to restraint since after habituation they respond to it with neither stress fever^59^ nor other signs of stress^60–62^.

All procedures were conducted under protocols approved by the Institutional Animal Use and Care Committee of the University of Pecs (registration no.: BA02/2000-6/2018) and were in accordance with the directives of the National Ethical Council for Animal Research and those of the European Communities Council. The study is reported in compliance with ARRIVE guidelines.

### Anesthesia and perioperative care

Mice were anesthetized via i.p. injection of a ketamine-xylazine cocktail (81.7 and 9.3 mg/kg, respectively) and received antibiotic prophylaxis with gentamicin (6 mg/kg) intramuscularly. To prevent intra- and postoperative hypothermia, mice were kept on a heating pad (model V500DVstat; PECO Services Ltd., Brough, UK) during the surgery, and then they were allowed to recover from anesthesia in a temperature-controlled chamber (model BJPX-B400II; Biobase; Jinan, China) set to an ambient temperature of 28 °C. Mice were allowed to recover for 4 days before the experiment.

### Intraperitoneal catheter implantation

To minimize stress during substance administration in the experiment, a polyethylene (PE)-50 catheter filled with pyrogen-free saline was implanted into the peritoneal cavity of each mouse, similarly as in previous studies^57,58^. In brief, through a small midline incision on the abdominal wall, the internal end of the catheter was fixed to the left side of the abdominal wall with a suture, while the external end of the catheter was tunneled under the skin to the nape, where it was exteriorized and heat-sealed. The surgical wound was sutured in layers. The catheter was flushed with 0.1 ml of saline on the day after the surgery and every other day thereafter.

### Experimental setup

The mice were placed in the thermocouple thermometry setup in cylindrical confiners and equipped with copper-constantan thermocouples (Omega Engineering, Stamford, CT, USA) to measure colonic temperature, a form of T_b_. The colonic thermocouple was inserted beyond the anal sphincter (3 cm deep); fixed to the base of the tail with adhesive tape; and plugged into a data logger device (Cole-Palmer, Vernon Hills, IL, USA) connected to a computer. Animals in their confiners were then placed into a temperature-controlled incubator (model MIDI F230S; PL Maschine Ltd., Tarnok, Hungary) or into a biochemistry incubator (model BJPX-Newark; Biobase; Jinan, China), in which the ambient temperature was set to 33 °C, which was thermoneutral for mice in these setups. In restrained mice in our setups, the thermoneutral zone is 31–33 °C^16,57^, while 30 °C is slightly below the zone^63^. The pre-implanted i.p. catheter was connected to a PE-50 extension, which was prefilled with the substance of interest and connected to a syringe placed in an infusion pump (model 975; Harvard Apparatus Inc., Holliston, MA, USA). The thermocouple thermometry setup has been extensively used by our group and has allowed us to study the dynamics of the fever response in rats and mice^15,16,64^.

### Substance administration

LPS from *Escherichia coli* 0111:B4 was purchased from Sigma-Aldrich (St. Louis, MO, USA). A stock solution of LPS (5 mg/ml) in pyrogen-free saline was stored at −20 °C. On the day of the experiment, the stock was diluted to a final concentration of 36 µg/ml. The diluted LPS solution or saline (for control animals) was infused (26 µl/min for 4 min) through the extension of the i.p. catheter to deliver LPS at a final dose of 120 µg/kg. Deep T_b_ was monitored for 6 h after the infusion. Administration of the substances was carried out between 10:00 a.m. and 11:30 a.m. in the experiments. After the experiment, the animals were euthanized with sodium pentobarbital (100 mg/kg, i.p.).

### Molecular biology

#### Tissue harvesting

On the day of the experiment, each mouse was placed in a confiner and left to acclimate in the incubator for ~ 2 h and then infused with LPS or saline as in the thermophysiological experiments. The time point for tissue harvesting was chosen based on the T_b_ curves. We collected blood and tissues at the time when we observed the biggest difference in deep T_b_ between the LPS-treated *Pacap*^*-/-*^ and *Pacap*^+*/*+^ mice. At the 210-min time point after infusion, the mice were anesthetized with ketamine-xylazine cocktail through the i.p. catheter. Blood samples were collected from the left ventricle, transferred to an Eppendorf tube, and were allowed to clot for 25 min before centrifugation at 10000 rpm for 10 min, in a refrigerated centrifuge at 4 °C. Serum fractions were collected, pooled, and stored at −80 °C until use in the assay. For collection of lung, liver, and brain tissue samples for RT-qPCR, each mouse was transcardially perfused with 0.1 M phosphate-buffered saline. Samples of the liver and the lung were collected rapidly and snap frozen in liquid nitrogen. Then the entire brain was removed, the hypothalamus was dissected and frozen. All tissue samples were stored at −80 °C.

#### Measurement of serum IL-1α, IL-1β, IL-6, IL-10, and TNF-α cytokine concentrations

Luminex xMAP technology was used to determine the protein concentrations of IL-1α, IL-1β, IL-6, IL-10, and TNF-α cytokines performing Milliplex Mouse High Sensitivity T Cell Magnetic Bead Panel (catalog number: MHSTCMAG-70K, Merck KGaA, Darmstadt, Germany) according to the manufacturer’s instructions. Briefly, all samples were thawed and tested undiluted in a blind-fashion and in duplicate. All reagents of the kit were brought to room temperature before use. 50 µl volume of each sample, standard, and control was added to a 96-well plate (provided with the kit) containing 25 µl mix of capture antibody coated bead sets, each internally color-coded with fluorescent dyes. Following 16 h of incubation, biotinylated detection antibody and streptavidin-PE were added to the plate after 60 and 30 min incubation steps, respectively. After the last washing step, 150 µl drive fluid was added to the wells, the plate was incubated for an additional 5 min on a shaker and immediately read on the Luminex MAGPIX instrument. Luminex xPonent 4.2 software was used for data acquisition. Five-PL regression curve were generated to plot the standard curves for all analyte by the Belysa v1.1 (Merck Millipore, Darmstadt, Germany) software calculating with bead median fluorescence intensity values. Results are given in pg/ml.

#### RNA isolation and quantitative real-time polymerase chain reaction (RT-qPCR)

As in our previous studies^63,65^, total RNA was extracted from the lungs, liver, and whole hypothalamus of treated and untreated *Pacap*^+*/*+^ and *Pacap*^*-/-*^ mice. The extraction was performed using TRI Reagent (Molecular Research Center Inc., Cincinnati, OH) in combination with the Direct-Zol RNA Isolation Miniprep Kit (Zymo Research, Irvine, CA), following the manufacturer’s protocols. RNA samples were treated with deoxyribonuclease I (Zymo Research, Irvine, CA) and quantified by Jenway Genova Nano Micro-Volume Spectrophotometer (Thermo Fisher Scientific, Budapest, Hungary). 500 ng purified RNA was reverse transcribed into cDNA using Tetro cDNA Synthesis Kit (Meridian Bioscience, Memphis, Tennessee, USA). Real-time qPCR was conducted with Quantstudio 5 System using glyceraldehyde 3-phosphate dehydrogenase (GAPDH) as a reference gene. Each reaction contained 20 ng cDNA, 1 × Luminaris HiGreen Low ROX qPCR Master Mix (Thermo Fisher Scientific), and 0.3 µM from each primer. RT-qPCR cycle conditions were as follows: 95 °C for 10 min, followed by 40 cycles of 95 °C for 30 s, 60 °C for 30 s, then 72 °C for 1 min. The following primer pairs were used to amplify the target loci: GAPDH sense: 5’-TTCACCACCATGGAGAAG-3’ and antisense: 5’-GGCATGGACTGTGGTCATGA-3’. COX-2 sense: 5’-GGGTTGCTGGGGGAAGAAA-3’ and antisense: 5’- CTCTGCTCTGGTCAATGGAGG-3’. All reactions were carried out in triplicate, and the mean value of the threshold cycles (Ct) was used for the determination of mRNA expression levels. The relative gene expression ratios were calculated according to the comparative ΔΔCt method using samples of untreated animals as calibrator. Measurements included a dissociation curve analysis to verify the amplification specificity. During the gene expression calculations, primer efficiencies were considered^66^.

### Data processing and analysis

Data on deep T_b_, serum cytokine levels, and COX-2 expression were compared by two-way ANOVA, followed by Fisher’s LSD* post hoc* tests, as in our previous studies^17,63^. For statistical analysis, Sigmaplot 11.0 (Systat Software, San Jose, CA, USA) software was used. Differences were considered statistically significant when *p* < 0.05. All data are presented as mean ± SEM.

## Data Availability

All data generated or analyzed during this study are included in this published article.
